# Supplementary material for: Behind closed doors: Protective social behavior during the COVID-19 pandemic
Source: PLoS One. 2023 Jun 28;18(6):e0287589. doi: 10.1371/journal.pone.0287589 (PMC10306218; doi:10.1371/journal.pone.0287589)
Supplement: S1 Appendix — (DOCX) [file pone.0287589.s001.docx]

**S1 Appendix: Sample Description**

**Understanding America Study Panel:** The Understanding America Study (UAS) is a probability-based internet panel of approximately 9,500 non-institutionalized adults residing in the United States. UAS panelists are recruited via address-based sampling, which allows for valid statistical inferences and avoids the coverage problems of convenience web-based panels. All UAS surveys are conducted in English or Spanish and panelists are provided with internet-enabled tablets if needed. Survey respondents received $20 per 30 minutes of survey time.

From April 1, 2020, to July 6, 2021, 10,279 UAS panelists were invited to participate in a COVID-19 tracking survey called the “Understanding Coronavirus in America” survey^25^; 88% consented to participate. Consenting panelists were surveyed biweekly (from April 1, 2020, to February 16, 2021) or every four weeks (from February 17, 2021, to July 20, 2021) about a number of topics related to COVID-19, including their mental and physical health, preventive health behaviors, Each day, on a rolling basis, one-fourteenth (one-twenty-eighth after February 17, 2021) of the panel were invited to complete a given survey wave over the course of two weeks. The mean completion rate per wave was 75%. Each day, on a rolling basis, one-fourteenth (one-twenty-eighth after February 17, 2021) of the panel were invited to complete a given survey wave over the course of two weeks.

**Analytic Sample:** Our starting sample comprises 8,616 UAS panel members who participated in at least one wave of the Understanding Coronavirus in America survey and who answered questions about their private masking and social-distancing behavior. Among these respondents, 52% are female, 54% are married, 40% are Democrats, 27% are aged 18-34 years, 18% are aged 65+ years, 61% are non-Hispanic White, 18% are Hispanic, 12% are non-Hispanic Black, and 6% are non-Hispanic Asian. The socio-economic composition of the sample is 37% high school degree or less, 35% Bachelor’s degree or more, 28% household income of less than $30,000, and 23% household income of $100,000 or more. Below, select demographic characteristics are presented alongside population benchmarks from the 2020 ACS 5-Year Estimates. Note that regression model sample sizes vary according to the number of respondents who have complete data (i.e. non-missing values in at least one survey wave) for the outcomes and predictors of interest in a given model. Domain-specific regression model sample sizes (reported in S4 Appendix) range from 6,040 (socio-demographic characteristics model) to 8,229 (work context). The meta-model sample size is 5,364, which reflects the number of respondents with complete data on every measure.

|  | Analytic Sample | U.S. Adult Population, Age 18+ |
| --- | --- | --- |
|  | (N=8,616) | (N=253,272,570) |
| Age |  |  |
| 18-34 | 27.4% | 30.0% |
| 35-49 | 29.9% | 24.4% |
| 50-64 | 24.7% | 24.9% |
| 65+ | 18.0% | 20.7% |
| Gender |  |  |
| Female | 52.23% | 51.3% |
| Male | 47.7% | 48.7% |
| Race/Ethnicity |  |  |
| White | 61.0% | 63.1% |
| Hispanic | 17.7% | 16.1% |
| Black | 11.8% | 12.3% |
| Asian | 5.6% | 5.8% |
| Other | 3.9% | 2.7% |
| Education |  |  |
| High School or Less | 37.1% | 38.9% |
| Some College | 28.4% | 30.7% |
| B.A. or More | 34.5% | 30.4% |
