# Supplementary material for: Behind closed doors: Protective social behavior during the COVID-19 pandemic
Source: PLoS One. 2023 Jun 28;18(6):e0287589. doi: 10.1371/journal.pone.0287589 (PMC10306218; doi:10.1371/journal.pone.0287589)
Supplement: S2 Appendix — (DOCX) [file pone.0287589.s002.docx]

**S2 Appendix: Select Measure Descriptions**

**Socio-Demographic Measures:**

To measure socio-demographic characteristics, we draw on indicators of race and ethnicity (non-Hispanic white, non-Hispanic Black, non-Hispanic Asian, Hispanic, and non-Hispanic other), gender (male, female), age (18-34 years, 35-49 years, 50-64 years, 65+ years), education (high school degree or less, some college, Bachelor’s degree or more), household income (less than $30,000, $30,000-59,999, $60,000-99,999, $100,000+), marital status (married, never married, other), at least one chronic health condition (diabetes, cancer (other than skin cancer), heart disease, high blood pressure, asthma, chronic lung disease, kidney disease, autoimmune disorder, mental health condition, obesity), and health insurance status (yes, no). All measures except the last two (chronic conditions and health insurance status) were collected and updated by UAS respondents on a quarterly basis through the UAS *My Household* survey. Measures of health insurance status and chronic health conditions were collected in waves 1-28 of the COVID tracking survey.

**Health Beliefs Measures:**

To measure perceived susceptibility, we use responses to three questions: “On a scale of 0 to 100 percent, what is the percent chance that you will get the coronavirus in the next three months? If you’re not sure, please give your best guess” (response option: 0% - 100% visual linear scale), “On a scale of 0 to 100 percent, what is the percent chance that someone who is vaccinated against the coronavirus could still catch it? If you’re not sure, please give your best guess” (response option: 0% - 100% visual linear scale), and “How safe is it to visit others in their home?” (response options: extremely safe, somewhat safe, somewhat unsafe, extremely unsafe, unsure). To measure perceived severity, we use responses to the question “If you get the coronavirus, what is the percent chance you will die from it? If you’re not sure, please give your best guess” (response option: 0% - 100% visual linear scale).

To measure perceived benefits associated with protective behavior, we rely on respondents’ self-reported agreement with the following statements about face masks: “Wearing a mask helps keep me safe from coronavirus,” “Wearing a mask keeps others safe from the coronavirus,” “Wearing a mask is not needed because I am not infected”, “Wearing a mask is not needed when I am with other people who are healthy,” and “I keep enough distance so that I don’t need a mask.” Response options included strongly agree, agree, neither agree nor disagree, disagree, strongly disagree. Where needed, responses were reverse-coded for our analysis. To measure perceived barriers to protective behavior, we rely on respondents’ self-reported agreement with these statements about face masks: “Wearing a mask is dangerous to my health,” “Others may feel threatened if I cover my face,” “I would like to wear a mask, but I cannot afford to buy one,” and “A mask is too uncomfortable to wear.” To measure perceived self-efficacy, we rely on self-reported frequency of feeling “Unable to control the important things in your life,” from the perceived stress scale.
