# Supplementary material for: Behind closed doors: Protective social behavior during the COVID-19 pandemic
Source: PLoS One. 2023 Jun 28;18(6):e0287589. doi: 10.1371/journal.pone.0287589 (PMC10306218; doi:10.1371/journal.pone.0287589)
Supplement: S3 Appendix — (DOCX) [file pone.0287589.s003.docx]

**S4 Appendix: Regression Models**

**Socio-Demographic Factors (C=0.64; N=6,040)**

| Effect | OR (95% CI) | P |
| --- | --- | --- |
| Race/Ethnicity (Ref = White) |  | **<0.001** |
| Hispanic | 2.25 (1.95, 2.60) | **<0.001** |
| Black | 3.28 (2.76, 3.90) | **<0.001** |
| Asian | 2.47 (1.96, 3.10) | **<0.001** |
| Other | 1.15 (0.90, 1.49) | 0.271 |
| Gender (Ref = Female) |  | **0.003** |
| Male | 0.86 (0.78, 0.95) | **0.003** |
| Age (Ref = 18-34) |  | **<0.001** |
| 35-49 | 1.12 (0.97, 1.30) | 0.111 |
| 50-64 | 1.85 (1.58, 2.15) | **<0.001** |
| 65+ | 2.62 (2.21, 3.12) | **<0.001** |
| Education (Ref = High School or Less) |  | **<0.001** |
| Some College | 1.21 (1.06, 1.36) | **0.003** |
| Bachelor's Degree or More | 1.55 (1.36, 1.77) | **<0.001** |
| Household Income (Ref = Less than $30,000) |  | **<0.001** |
| $30,000-$59,999 | 0.69 (0.60, 0.79) | **<0.001** |
| $60,000-$99,999 | 0.58 (0.50, 0.68) | **<0.001** |
| $100,000 or more | 0.62 (0.53, 0.74) | **<0.001** |
| Marital Status (Ref = Married) |  | **0.002** |
| Never Married | 1.17 (1.02, 1.35) | **0.025** |
| Other | 0.87 (0.76, 1.00) | **0.042** |
| At Least 1 Chronic Condition | 1.03 (0.93, 1.15) | 0.520 |
| Health Insurance | 1.88 (1.58, 2.22) | **<0.001** |

**Political Affiliation (C=0.64; N=6,100)**

| Effect | OR (95% CI) | P |
| --- | --- | --- |
| Political Affiliation (Ref = Democrat) |  | **<0.001** |
| Other | 0.65 (0.57, 0.74) | **<0.001** |
| Republican | 0.26 (0.23, 0.29) | **<0.001** |

**Health Beliefs (C=0.80; N=7,579)**

| Effect | OR (95% CI) | P |
| --- | --- | --- |
| Perceived Risk of Infection (+10%) | 0.79 (0.76, 0.82) | **<0.001** |
| Perceived Risk of Death (+10%) | 1.17 (1.14, 1.20) | **<0.001** |
| Mask Keeps Me Safe | 1.24 (1.11, 1.38) | **<0.001** |
| Mask Keeps Others Safe | 0.97 (0.84, 1.11) | 0.622 |
| % Chance of Getting COVID (+10%) | 1.03 (1.00, 1.05) | **0.033** |
| Mask Dangerous to Health | 1.04 (0.94, 1.16) | 0.453 |
| Mask Is Uncomfortable | 0.94 (0.87, 1.01) | 0.100 |
| Mask Not Needed because Not Infected | 0.88 (0.72, 1.07) | 0.201 |
| Mask Not Needed for Healthy People | 0.48 (0.42, 0.54) | **<0.001** |
| Mask Not Needed because Keep Distance | 0.93 (0.83, 1.03) | 0.157 |
| Mask Not Needed because COVID Is Not a Threat | 1.10 (0.93, 1.29) | 0.280 |
| Mask Makes Others Feel Threatened | 0.92 (0.84, 1.02) | 0.102 |
| Cannot Afford Mask | 2.29 (2.05, 2.56) | **<0.001** |
| Perceived Safety of Visiting Other Households | 2.81 (2.57, 3.06) | **<0.001** |
| Unable to Control Important Things in Life | 0.88 (0.83, 0.93) | **<0.001** |

**Informational Trust (C=0.64; N=8,062)**

| Effect | OR (95% CI) | P |
| --- | --- | --- |
| Trust in National Mainstream Media | 1.30 (1.04, 1.62) | **0.020** |
| Trust in National Left-Leaning Media | 1.86 (1.55, 2.23) | **<0.001** |
| Trust in National Right-Leaning Media | 0.91 (0.85, 0.98) | **0.011** |
| Trust in Local Media | 0.92 (0.78, 1.07) | 0.277 |
| Trust in CDC, HHS | 1.11 (0.98, 1.24) | 0.092 |
| Trust in Physician | 1.04 (0.96, 1.14) | 0.343 |
| Trust in Local Public Health Officials | 0.87 (0.76, 0.99) | **0.039** |
| Trust in Social Media | 1.13 (1.01, 1.27) | **0.039** |
| Trust in Family/Friends as Information Source | 0.53 (0.48, 0.60) | **<0.001** |

**Interpersonal Context (C=0.59; N=8,080)**

| Effect | OR (95% CI) | P |
| --- | --- | --- |
| Elderly in Household | 1.44 (1.32, 1.59) | **<0.001** |
| Children Under 5 | 0.83 (0.72, 0.96) | **0.012** |
| Children 5-18 | 1.07 (0.94, 1.20) | 0.315 |
| Number of Closer Friends/Family (Ref = <10) |  | **<0.001** |
| 10-20 | 0.58 (0.52, 0.65) | **<0.001** |
| 20-37 | 0.42 (0.37, 0.47) | **<0.001** |
| 38+ | 0.41 (0.36, 0.46) | **<0.001** |
| Perceived Everyday Discrimination | 0.87 (0.74, 1.01) | 0.069 |
| Perceived COVID Discrimination | 0.71 (0.52, 0.95) | **0.023** |

**Work Context (C=0.58; N=8,229)**

| Effect | OR (95% CI) | P |
| --- | --- | --- |
| Work In-Person | 0.45 (0.39, 0.51) | **<0.001** |
| Hours Worked in Past Week | 1.00 (1.00, 1.00) | 0.240 |

**Community Context (C=0.62; N=8,087)**

| Effect | OR (95% CI) | P |
| --- | --- | --- |
| COVID Deaths per Capita | 1.00 (0.98, 1.02) | 0.983 |
| 2020 Unemployment Rate | 1.05 (1.03, 1.07) | **<0.001** |
| Neighborhood Disorder | 1.15 (1.07, 1.23) | **<0.001** |
| % Voted for Trump | 0.87 (0.85, 0.89) | **<0.001** |

**Meta-Regression Model (C=0.82; N=5,364)**

| Effect | OR (95% CI) | P |
| --- | --- | --- |
| Socio-Demographics: Higher-Adherence Odds (x2) | 1.23 (1.14, 1.32) | **<0.001** |
| Political Affiliation: Higher-Adherence Odds (x2) | 1.06 (0.98, 1.16) | 0.166 |
| Health Beliefs: Higher-Adherence Odds (x2) | 1.94 (1.87, 2.02) | **<0.001** |
| Informational Trust: Higher-Adherence Odds (x2) | 1.00 (0.91, 1.09) | 0.992 |
| Interpersonal Context: Higher-Adherence Odds (x2) | 1.87 (1.69, 2.07) | **<0.001** |
| Work Context: Higher-Adherence Odds (x2) | 1.34 (1.20, 1.50) | **<0.001** |
| Community Context: Higher-Adherence Odds (x2) | 1.52 (1.38, 1.69) | **<0.001** |

*Note:* To assess the relative performance of each domain in predicting high adherence, we evaluated how much a two-fold increase in each domain’s predicted odds actually increased the odds of adherence after controlling for other domains. In this model, all domains are adjusted for and each predictor represents a specific domain’s predictive value – that is, the predicted probability of high adherence associated with the modification of that domain.
