# Supplementary figures and images for: Behind closed doors: Protective social behavior during the COVID-19 pandemic
Source: PLoS One. 2023 Jun 28;18(6):e0287589. doi: 10.1371/journal.pone.0287589 (PMC10306218; doi:10.1371/journal.pone.0287589)

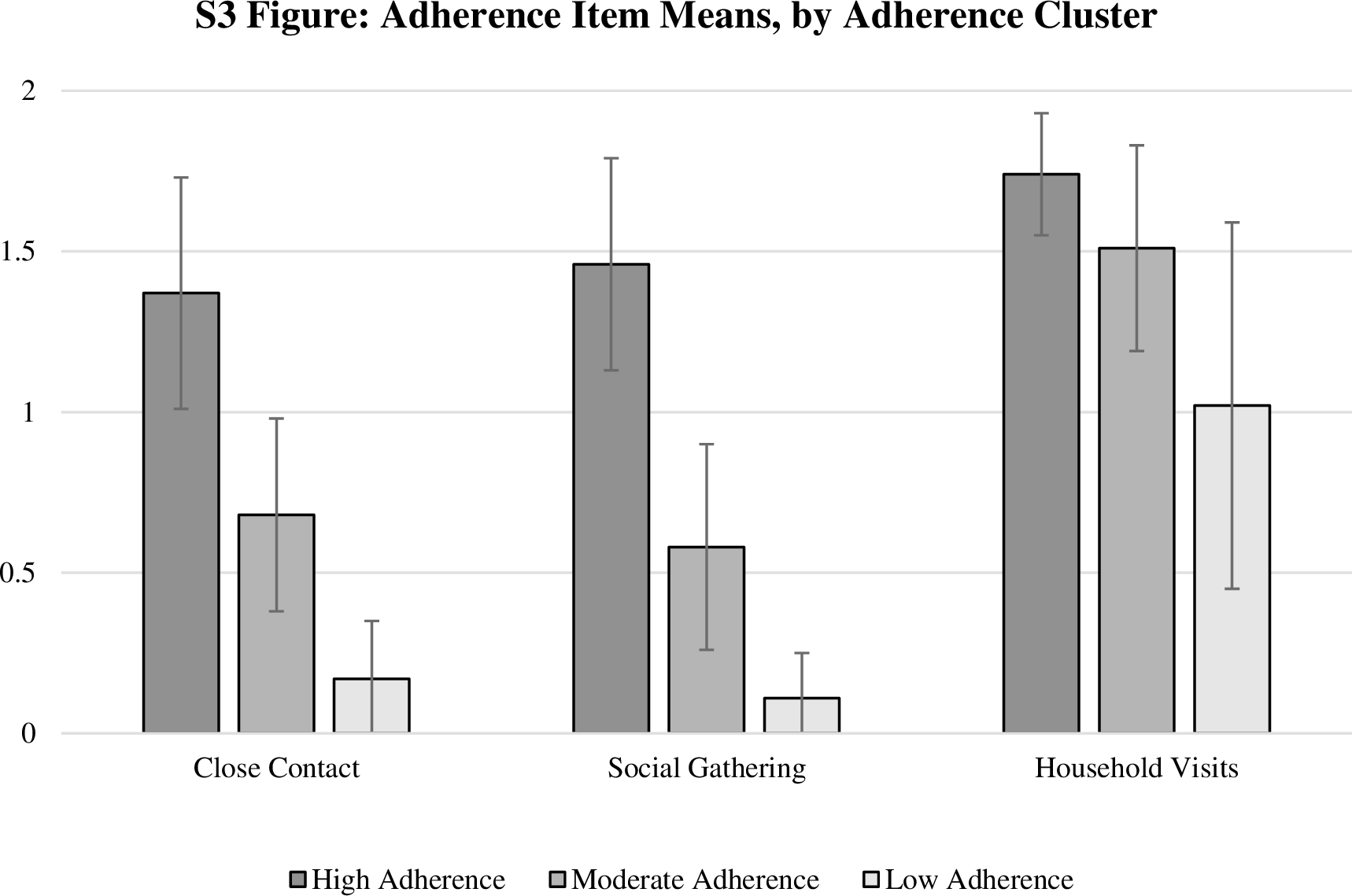

Supplement: S1 Fig — (TIF) [file pone.0287589.s004.tif]
