# Supplementary material for: Behind closed doors: Protective social behavior during the COVID-19 pandemic
Source: PLoS One. 2023 Jun 28;18(6):e0287589. doi: 10.1371/journal.pone.0287589 (PMC10306218; doi:10.1371/journal.pone.0287589)
Supplement: Attachment — Submitted filename: PONE-D-22-23931_reviewer (reviewed).pdf [file pone.0287589.s005.pdf]

PLOS ONE

Behind Closed Doors: Protective Social Behavior During the COVID-19 Pandemic

--Manuscript Draft--

**Manuscript Number:**	PONE-D-22-23931
**Article Type:**	Research Article
**Full Title:**	Behind Closed Doors: Protective Social Behavior During the COVID-19 Pandemic
**Short Title:**	Protective Social Behavior During the COVID-19 Pandemic
**Corresponding Author:**	Kyla Thomas<br>University of Southern California<br>Los Angeles, UNITED STATES
**Keywords:**	protective behavior; social-distancing; masking; COVID-19; pandemic; health beliefs model
**Abstract:**	The success of personal non-pharmaceutical interventions as a public health strategy requires a high level of compliance from individuals in private social settings. Strategies to increase compliance in these hard-to-reach settings depend upon a comprehensive understanding of the patterns and predictors of protective social behavior. Social cognitive models of protective behavior emphasize the contribution of individual-level factors while social-ecological models emphasize the contribution of environmental factors. This study draws on 28 waves of survey data from the Understanding Coronavirus in America survey to measure patterns of adherence to two protective social behaviors – private social-distancing behavior and private masking behavior – during the COVID-19 pandemic and to assess the role individual and environmental factors play in predicting adherence. Results show that patterns of adherence fall into three categories marked by high, moderate, and low levels of protective behavior, with just under half of respondents exhibiting a high level of adherence. Health beliefs emerge as the single strongest predictor of adherence. All other environmental and individual-level predictors have relatively poor predictive power or primarily indirect effects.
**Order of Authors:**	Kyla Thomas, PhD
	Peter Szilagyi, MD, MPH
	Sitaram Vangala, MS
	Rebecca Dudovitz, MD, MSHS
	Megha Shah, MD, MPH, MS
	Nathalie Vizueta, PhD
	Arie Kapteyn, PhD
**Additional Information:**	
**Question**	**Response**
**Financial Disclosure**<br><br>Enter a financial disclosure statement that describes the sources of funding for the work included in this submission. Review the submission guidelines for detailed requirements. View published research articles from *PLOS ONE* for specific examples.<br><br>This statement is required for submission and **will appear in the published article** if	Data collection for this study was supported by the University of Southern California, the Bill & Melinda Gates Foundation, and federal funds from the National Institute on Aging (grant 5U01AG054580-03) and the National Science Foundation (grant 2028683). The sponsors had no role in study design, data collection and analysis, decision to publish, or preparation of the manuscript.

Powered by Editorial Manager® and ProduXion Manager® from Aries Systems Corporation

the submission is accepted. Please make sure it is accurate.<br><br>**Unfunded studies**<br>Enter: *The author(s) received no specific funding for this work.*<br><br>**Funded studies**<br>Enter a statement with the following details:<br>• Initials of the authors who received each award<br>• Grant numbers awarded to each author<br>• The full name of each funder<br>• URL of each funder website<br>• Did the sponsors or funders play any role in the study design, data collection and analysis, decision to publish, or preparation of the manuscript?<br>• **NO** - Include this sentence at the end of your statement: *The funders had no role in study design, data collection and analysis, decision to publish, or preparation of the manuscript.*<br>• **YES** - Specify the role(s) played.<br><br>* typeset	
**Competing Interests**<br><br>Use the instructions below to enter a competing interest statement for this submission. On behalf of all authors, disclose any competing interests that could be perceived to bias this work—acknowledging all financial support and any other relevant financial or non-financial competing interests.<br><br>This statement is required for submission and **will appear in the published article** if the submission is accepted. Please make sure it is accurate and that any funding sources listed in your Funding Information later in the submission form are also declared in your Financial Disclosure statement.<br><br>View published research articles from *PLOS ONE* for specific examples.	The authors have declared that no competing interests exist.

**NO authors have competing interests**<br><br>Enter: *The authors have declared that no competing interests exist.*<br><br>**Authors with competing interests**<br><br>Enter competing interest details beginning with this statement:<br><br>*I have read the journal's policy and the authors of this manuscript have the following competing interests: [insert competing interests here]*<br><br>* typeset	
**Ethics Statement**<br><br>Enter an ethics statement for this submission. This statement is required if the study involved:<br><br>• Human participants<br>• Human specimens or tissue<br>• Vertebrate animals or cephalopods<br>• Vertebrate embryos or tissues<br>• Field research<br><br>Write "N/A" if the submission does not require an ethics statement.<br><br>General guidance is provided below. Consult the submission guidelines for detailed instructions. **Make sure that all information entered here is included in the Methods section of the manuscript.**	Data collection was approved by the University of Southern California Institutional Review Board. Survey respondents completed a consent form online prior to their participation in the Understanding Coronavirus in America survey.

Powered by Editorial Manager® and ProduXion Manager® from Aries Systems Corporation

**Format for specific study types**<br><br>**Human Subject Research (involving human participants and/or tissue)**<br>• Give the name of the institutional review board or ethics committee that approved the study<br>• Include the approval number and/or a statement indicating approval of this research<br>• Indicate the form of consent obtained (written/oral) or the reason that consent was not obtained (e.g. the data were analyzed anonymously)<br><br>**Animal Research (involving vertebrate animals, embryos or tissues)**<br>• Provide the name of the Institutional Animal Care and Use Committee (IACUC) or other relevant ethics board that reviewed the study protocol, and indicate whether they approved this research or granted a formal waiver of ethical approval<br>• Include an approval number if one was obtained<br>• If the study involved *non-human primates*, add *additional details* about animal welfare and steps taken to ameliorate suffering<br>• If anesthesia, euthanasia, or any kind of animal sacrifice is part of the study, include briefly which substances and/or methods were applied<br><br>**Field Research**<br><br>Include the following details if this study involves the collection of plant, animal, or other materials from a natural setting:<br>• Field permit number<br>• Name of the institution or relevant body that granted permission	
**Data Availability**<br><br>Authors are required to make all data underlying the findings described fully available, without restriction, and from the time of publication. PLOS allows rare exceptions to address legal and ethical concerns. See the PLOS Data Policy and FAQ for detailed information.	No - some restrictions will apply

A Data Availability Statement describing where the data can be found is required at submission. Your answers to this question constitute the Data Availability Statement and **will be published in the article**, if accepted.<br><br>**Important:** Stating 'data available on request from the author' is not sufficient. If your data are only available upon request, select 'No' for the first question and explain your exceptional situation in the text box.<br><br>Do the authors confirm that all data underlying the findings described in their manuscript are fully available without restriction?	
**Describe where the data may be found in full sentences. If you are copying our sample text, replace any instances of XXX with the appropriate details.**<br><br>• If the data are **held or will be held in a public repository**, include URLs, accession numbers or DOIs. If this information will only be available after acceptance, indicate this by ticking the box below. For example: *All XXX files are available from the XXX database (accession number(s) XXX, XXX.).*<br>• If the data are all contained **within the manuscript and/or Supporting Information files**, enter the following: *All relevant data are within the manuscript and its Supporting Information files.*<br>• If neither of these applies but you are able to provide **details of access elsewhere**, with or without limitations, please do so. For example:<br><br>*Data cannot be shared publicly because of [XXX]. Data are available from the XXX Institutional Data Access / Ethics Committee (contact via XXX) for researchers who meet the criteria for access to confidential data.*<br><br>*The data underlying the results presented in the study are available from (include the name of the third party*	All Understanding America Study (UAS) survey data files are available on the Understanding America Study website (https://uasdata.usc.edu/index.php), pending submission and approval of a DUA. All external, county-level data sources are publicly available for download via the New York Times (https://www.nytimes.com/interactive/2021/us/covid-cases.html), Bureau of Labor Statistics (https://www.bls.gov/lau/#cntyaa), and MIT (https://electionlab.mit.edu/data). A linked file in which external data sources are linked to UAS survey data cannot be shared publicly to preserve the anonymity of the respondents. However, this file can be made available to researchers who meet the criteria for access to confidential data (contact uas-l@usc.edu for more information).

and contact information or URL).<br>• This text is appropriate if the data are owned by a third party and authors do not have permission to share the data.<br><br>* typeset	
Additional data availability information:	

Powered by Editorial Manager® and ProduXion Manager® from Aries Systems Corporation

Manuscript

Click here to access/download;Manuscript;ProtectiveBehavior_plosone.docx

Behind Closed Doors:
Protective Social Behavior during the COVID-19 Pandemic

Kyla Thomas, PhD[1], Peter G. Szilagyi, MD, MPH[2], Sitaram Vangala, MS[3], Rebecca N. Dudovitz, MD, MSHS[2], Megha D. Shah, MD, MPH, MS[4], Nathalie Vizueta, PhD[2], Arie Kapteyn, PhD[1]

1. Center for Economic and Social Research, Dornsife College of Letters Arts and Sciences, University of Southern California, 635 Downey Way, VPD, Los Angeles, CA 90089.
2. Department of Pediatrics, David Geffen School of Medicine, University of California at Los Angeles, 10833 Le Conte Avenue, Los Angeles, CA 90095.
3. Department of Medicine Statistics Core, David Geffen School of Medicine, University of California at Los Angeles, 1100 Glendon Avenue, Los Angeles, CA 90024
4. Los Angeles County Department of Public Health, Office of Health Assessment and Epidemiology, 313 N. Figueroa Street, Los Angeles, CA 90012

Corresponding Author: Kyla Thomas; Center for Economic and Social Research, Dornsife College of Letters Arts and Sciences, University of Southern California, 635 Downey Way, VPD, Los Angeles, CA 90089; Email: kylathom@usc.edu; Phone: 213-821-8923.

Behind Closed Doors:
Protective Social Behavior during the COVID-19 Pandemic

Abstract

The success of personal non-pharmaceutical interventions as a public health strategy requires a high level of compliance from individuals in private social settings. Strategies to increase compliance in these hard-to-reach settings depend upon a comprehensive understanding of the patterns and predictors of protective social behavior. Social cognitive models of protective behavior emphasize the contribution of individual-level factors while social-ecological models emphasize the contribution of environmental factors. This study draws on 28 waves of survey data from the Understanding Coronavirus in America survey to measure patterns of adherence to two protective social behaviors – private social-distancing behavior and private masking behavior – during the COVID-19 pandemic and to assess the role individual and environmental factors play in predicting adherence. Results show that patterns of adherence fall into three categories marked by high, moderate, and low levels of protective behavior, with just under half of respondents exhibiting a high level of adherence. Health beliefs emerge as the single strongest predictor of adherence. All other environmental and individual-level predictors have relatively poor predictive power or primarily indirect effects.

Behind Closed Doors:
Protective Social Behavior during the COVID-19 Pandemic

Introduction

Mitigating risk for future pandemics will depend upon the immediate and widespread adoption of personal non-pharmaceutical interventions (NPIs) including social-distancing and masking. The challenge of personal NPIs as a public health strategy is that they require sustained behavioral compliance on the part of individuals. Lockdowns and mask mandates ensure some compliance in public settings but restrictions are not always accepted or enforced, especially in private social settings where risk of infection is high[1] but mechanisms of enforcement are largely absent.[2] Anticipating infection risk and designing effective interventions to increase behavioral compliance in hard-to-reach settings requires a comprehensive understanding of the patterns and predictors of protective social behavior.

Current models of protective behavior fall into two general groups: social-cognitive and social-ecological models. Social-cognitive models emphasize the contribution of individual or intrapersonal factors. One of the most widely-used of these models – the Health Beliefs Model (HBM) – focuses on the role of attitudes and beliefs.[3,4] Studies show that perceptions of infection risk, fear of disease, self-efficacy, and belief in the effectiveness of NPIs are positively associated with adherence to protective behavior.[5–10] Beyond health beliefs, studies have also identified political ideology[11,12] and trusted information sources[13–15] as intrapersonal factors associated with preventive behavior.

In practice, however, attitudes and beliefs tend to predict behavioral *intention* better than they do behavior, and intention tends to explain only a portion of the variance observed in health-related behavior.[16,17] Social-ecological models suggest environmental factors may fill this

explanatory gap. Environmental factors are not entirely absent from social-cognitive models but they have limited explanatory power and rarely receive the same theoretical or empirical attention as health beliefs. The Social-Ecological Model (SEM), in contrast, attributes preventive behavior to the interplay between individuals and the wide range of interpersonal (i.e. family, friend), organizational (i.e. work, healthcare, school), and community (i.e. social, political) contexts in which they are embedded[18–20]

Together, the HBM, SEM, and related studies point to a range of predictive factors and targets for behavioral intervention, from personal beliefs to the social, economic, and political conditions in which people live and work. When time and resources are limited, an effective pandemic response hinges on the ability of policymakers to target the factors that matter most. With respect to this question, however, a few gaps in the literature remain. First, the majority of studies examine protective behavior at a single point in time, or they assess the predictors of protective-behavioral change between time points. While valuable for the design of policy interventions, these studies offer limited insight into the *general* and *enduring* predictors of protective behavior. Second, most studies take a variable-centered, versus person-centered, approach to measuring protective behavior. They assume behaviors like masking and social distancing relate to one another in a uniform way across the population.[21] Third, most studies offer limited insight into the relative contribution of individual and environmental factors to protective behavior, especially in private social settings.[20]

The primary aim of this study is thus to offer a comprehensive assessment of protective social behavior during the COVID-19 pandemic. Drawing on 28 waves of survey data collected by the Understanding America Study from April 2020 to July 2021, we use cluster analysis to

derive patterns of adherence to private social-distancing and masking behavior. We then use meta-regression analysis to summarize the relative contribution of individual and environmental predictors to the prediction of adherence. Results suggest health beliefs are strongest direct predictor of adherence. Other predictors have relatively poor predictive power or primarily indirect effects.

Methods

Study Sample: The Understanding America Study (UAS) is a probability-based internet panel of approximately 9,500 non-institutionalized adults residing in the United States. UAS panelists are recruited via address-based sampling, which allows for valid statistical inferences and avoids the coverage problems of convenience web-based panels. All UAS surveys are conducted in English or Spanish and panelists are provided with internet-enabled tablets if needed. Survey respondents received $20 per 30 minutes of survey time.

From April 1, 2020, to July 6, 2021, 10,279 UAS panelists were invited to participate in a COVID-19 tracking survey called the "Understanding Coronavirus in America" survey[22]; 88% consented to participate. Consenting panelists were surveyed biweekly (from April 1, 2020, to February 16, 2021) or every four weeks (from February 17, 2021, to July 20, 2021) about a number of topics related to COVID-19, including their mental and physical health, preventive health behaviors. Each day, on a rolling basis, one-fourteenth (one-twenty-eighth after February 17, 2021) of the panel were invited to complete a given survey wave over the course of two weeks.

A total of 28 surveys were fielded, with a mean completion rate of 75%. Data collection was approved by the University of Southern California Institutional Review Board. All survey data are publicly available online. Analytic samples for this study comprise respondents with complete data (i.e. non-missing values in at least one survey wave) for our predictors and outcomes of interest.

Outcome Measures: Our outcome of interest is average adherence to private social-distancing and private masking behavior. In waves 1-28 of the COVID survey, respondents were asked if they had participated in 16 different social activities in the last seven days. Of the activities listed, we use responses to the following four to measure adherence to private social-distancing behavior: (1) "Gone to a friend, neighbor, or relative's residence (that is not your own)", (2) "Had visitors such as friends, neighbors or relatives are your residence," (3) "Attended a gathering with more than 10 people, such as a reunion, wedding, funeral, birthday party, or religious service," and (4) "Had close contact (within 6 feet) with people who do not live with you." We combined the first two items into a single item: "household visits." We refer to the third item as "social gatherings" and the fourth as "close contact."

A follow-up to the above was added to waves 7-28 to assess adherence to private masking behavior. For each activity that received a participation response of "Yes," respondents were asked "…how often, if ever, you wore a mask or face covering," with the following as response options: always, most of the time, rarely, never, unsure.

We applied the following coding scheme to measure adherence to private social-distancing and masking behavior. In waves 1-6, our coding scheme was dichotomous and based on social-distancing behavior. For each activity of interest (household visits, social gatherings,

close contact), respondents received a code of “1” (denoting adherence) if their participation response was “No” or “Unsure” and a code of “0” (denoting non-adherence) if their participation response was “Yes.”

In waves 7-28, our coding scheme was ordinal and based on both social-distancing and masking behavior. Respondents received a code of “2” (denoting high adherence) if their participation response was “No” or “Unsure”; they received a code of “1” (denoting moderate adherence) if their participation response was “Yes” AND their mask frequency response was “Always” or “Most of the time”; they received a code of “0” (denoting non-adherence) if their participation was “Yes” AND their mask frequency response was “Sometimes,” “Rarely,” “Never,” or “Unsure.”

After coding adherence, we performed a cluster analysis to group respondents on the basis of their adherence values across all 28 survey waves. Hierarchical complete linkage clustering was used, with a distance matrix derived from Euclidean distances along the first principal component of the time-averaged adherence measures. A 3-cluster solution was extracted, with one cluster consistently having the highest adherence measure means (“high adherence”), one consistently having the lowest (“low adherence”), and one consistently in between (“moderate adherence”).

Individual-Level Predictors: The conceptual framework for this study is presented in Figure 1. Our individual-level predictor domains include socio-demographic characteristics, political affiliation, trusted information sources, and health beliefs.

Fig 1: Modified Conceptual Framework and Measures, Based on Social-Ecological and Social-Cognitive Models

To measure socio-demographic characteristics, we draw on indicators of race and ethnicity (non-Hispanic white, non-Hispanic Black, non-Hispanic Asian, Hispanic, and non-Hispanic other), gender (male, female), age (18-34 years, 35-49 years, 50-64 years, 65+ years), education (high school degree or less, some college, Bachelor's degree or more), household income (less than $30,000, $30,000-59,999, $60,000-99,999, $100,000+), marital status (married, never married, other), at least one chronic health condition (diabetes, cancer (other than skin cancer), heart disease, high blood pressure, asthma, chronic lung disease, kidney disease, autoimmune disorder, mental health condition, obesity), and health insurance status (yes, no). All measures except the last two (chronic conditions and health insurance status) were collected and updated by UAS respondents on a quarterly basis through the UAS *My Household* survey. Measures of health insurance status and chronic health conditions were collected in waves 1-28 of the COVID tracking survey.

To measure political affiliation, we use the political party affiliation (Democrat, Republican, and Other) reported by respondents in UAS 318, a post-election poll distributed to full UAS panel from November 4, 2020, to December 15, 2020 (81% survey completion rate).

To measure trusted information sources, we rely on a question series included in waves 1, 7, 19-28 of the COVID survey. Respondents were asked to rate their level of trust in 22 sources of information about COVID-19, which we grouped into the following categories: national mainstream media, national left-leaning media, national right-leaning media, local media, CDC/HHS, physician, local public health officials, social media, and friends/family. To group sources, we averaged responses across information sources within a category and then averaged across waves.

To measure health beliefs, we draw on the following indicators, collected in waves 1-28 of the COVID survey unless otherwise noted: (1) perceived susceptibility, measured as perceived chance of getting COVID, perceived chance of vaccinated individuals getting COVID (waves 19-28), and perceived safety of visiting others in home, (2) perceived severity, measured as perceived chance of death if infected with COVID, (3) perceived benefits, measured as self-reported (dis)agreement that face masks are important or effective (waves 7, 9-28), (4) perceived barriers, measured as self-reported (dis)agreement that face masks are dangerous, threatening to others, unaffordable, uncomfortable (waves 7, 9-28), and (5) perceived self-efficacy, measured as self-reported frequency of feeling unable to control important things in life. See S1 Appendix for a full description of these measures.

Environmental Predictors: Our environmental predictor domains of interest are interpersonal context, work context, and community context. To measure interpersonal context, we draw on indicators of household composition (household members age 65+, children age 0-4, children age 5-18 – from the UAS *My Household* survey), number of close friends/family (<10, 10-20, 21-37, 38+ — from waves 1-5, 7, 9-28 of the COVID survey), self-reported experiences of everyday discrimination (everyday discrimination scale, short version[23], collected in waves 6-7, 9-28), and self-reported experiences of COVID-associated discrimination (adapted everyday discrimination, collected in waves 1-5, 7, 9-28). To measure work context, we rely on indicators of respondents' work conditions, including the number of hours worked in the past week and whether or not they worked in-person for at least one day in the last week, collected in waves 1-28. Non-workers received a value of "0" for these measures.

Lastly, to measure community context, we draw on measures of perceived neighborhood disorder ((dis)agreement with statements about neighborhood cleanliness, crime, vandalism, loitering – collected in waves 1-28 of the COVID survey), county-level administrative indicators of the COVID death rate (county-level deciles of COVID deaths per capita, published by the *New York Times*), unemployment rate (county-level 2020 unemployment rate, published by the *Bureau of Labor Statistics*), and support for presidential candidate Donald Trump (county-level deciles of % votes for Donald Trump in 2020 presidential election, published by *MIT)*.

Statistical Analysis: Our analytic strategy proceeded in three phases. First, we performed a cluster analysis to group respondents on the basis of their time-invariant patterns of self-reported adherence to private social-distancing and masking behavior. Second, we used ordinal logistic regression models to estimate the predictive value of individual predictors with respect to cluster membership, fitting separate models for each of the following predictor domains.

The predictive performance of each model is summarized in terms of concordance *C* statistics, which can be interpreted as the proportion of respondent pairs correctly ranked by the model in terms of adherence. Finally, we fitted a meta-regression model of cluster membership, representing each domain by the predicted probabilities of high adherence derived from the corresponding model. Ordinal logistic regression was used for the meta-regression analysis, with domain-specific predicted probabilities modeled on the log odds scale. All analyses accounted for survey sampling weights, and used two-sided 0.05 significance levels (SAS v. 9.4).

Results

Sample Description

Our starting sample comprises 8,616 UAS panel members who participated in at least one wave of the Understanding Coronavirus in America survey and who answered questions about their private masking and social-distancing behavior. Among these respondents, 52% are female, 53% are married, 40% are Democrats, 27% are aged 18-34 years, 18% are aged 65+ years, 61% are non-Hispanic White, 18% are Hispanic, 11% are non-Hispanic Black, and 6% are non-Hispanic Asian. The socio-economic composition of the sample is 37% high school degree or less, 35% Bachelor's degree or more, 28% household income of less than $30,000, and 23% household income of $100,000 or more.

Regression model sample sizes vary according to the number of respondents who have complete data (i.e. non-missing values in at least one survey wave) for the outcomes and predictors of interest in a given model. Domain-specific regression model sample sizes (reported in S3 Appendix) range from 6,040 (socio-demographic characteristics model) to 8,229 (work context). The meta-model sample size is 5,364, which reflects the number of respondents with complete data on every measure.

Patterns of Protective Social Behavior

The results of our cluster analysis reveal three distinct patterns of adherence to protective behavior in private settings: high adherence (44% of respondents), moderate adherence (38%), and low adherence (18%) behavior. Mean scores for each activity and cluster can be found in S2 Table.

As shown in Table 1, demographic characteristics are strongly related to cluster membership. Respondents who are under age 65, male, non-Hispanic white, high-income, lower education, or Republican are more likely to be classified as low adherence than respondents who are aged 65+, female, Hispanic or non-white, low-income, higher educated, or a Democrat, respectively.

Table 1. Demographic Summary by Adherence Cluster

	High Compliance (N=3,766)	Moderate Compliance (N=3,287)	Low Compliance (N=1,563)	P
Age				<0.001
18-34	923 (24.5%)	938 (28.5%)	502 (32.2%)	
35-49	1,004 (26.7%)	1,047 (31.9%)	521 (33.4%)	
50-64	1,025 (27.3%)	758 (23.1%)	342 (21.9%)	
65+	811 (21.6%)	544 (16.6%)	197 (12.6%)	
Gender				<0.001
Female	2,042 (54.2%)	1,712 (52.1%)	748 (47.8%)	
Male	1,724 (45.8%)	1,574 (47.9%)	815 (52.2%)	
Race/Ethnicity				<0.001
White	1,908 (50.7%)	2,125 (64.7%)	1,221 (78.1%)	
Hispanic	806 (21.4%)	511 (15.6%)	204 (13.0%)	
Black	650 (17.3%)	312 (9.5%)	57 (3.7%)	
Asian	262 (7.0%)	199 (6.1%)	17 (1.1%)	
Other	137 (3.6%)	136 (4.2%)	64 (4.1%)	
Education				<0.001
High School or Less	1,424 (37.8%)	1,161 (35.4%)	613 (39.2%)	
Some College	1,071 (28.4%)	906 (27.6%)	468 (29.9%)	
Bachelor's Degree or More	1,271 (33.8%)	1,216 (37.0%)	483 (30.9%)	
Household Income				<0.001
Less than $30,000	1,291 (34.3%)	811 (24.7%)	310 (19.9%)	
$30,000-$59,999	972 (25.9%)	886 (27.0%)	433 (27.7%)	
$60,000-$99,999	732 (19.5%)	796 (24.3%)	433 (27.7%)	
$100,000 or more	767 (20.4%)	787 (24.0%)	386 (24.7%)	
Marital Status				0.005
Married	1,946 (51.7%)	1,767 (53.8%)	898 (57.4%)	
Never Married	1,058 (28.1%)	898 (27.4%)	394 (25.2%)	
Other	760 (20.2%)	619 (18.8%)	272 (17.4%)	
Region				<0.001
Northeast	648 (17.3%)	590 (18.2%)	256 (16.4%)	
South	1,563 (41.8%)	1,178 (36.3%)	573 (36.7%)	

Midwest	594 (15.9%)	702 (21.6%)	427 (27.4%)	
West	931 (24.9%)	781 (24.0%)	305 (19.5%)	
Political Affiliation				<0.001
Democrat	1,388 (51.0%)	930 (39.4%)	146 (13.9%)	
Republican	632 (23.2%)	921 (39.1%)	671 (63.9%)	
Other	704 (25.8%)	508 (21.5%)	233 (22.2%)	

Predictors of Protective Social Behavior

The results of our ordinal logistic regression models are presented in Figure 2. Prediction models are ranked in order of their C-statistic, which summarizes the predictive performance of each model. Health beliefs are the strongest predictor of adherence to protective social behavior. With a C-statistic of 0.80, a model using health beliefs alone correctly ranks pairs of respondents in terms of adherence 80% of the time. All other predictors do a notably worse job of prediction, with individual-level domains emerging as only marginally more predictive than environmental domains.

Fig 2: Predictors of Adherence to Protective Social Behavior.

Note: C-statistics summarize the predictive performance of each prediction model. A C-statistic can be interpreted as the proportion of respondent pairs correctly ranked by the model in terms of adherence. For example, a C-statistic of 0.80 indicates the predictor domain correctly ranks pairs of respondents in terms of adherence 80% of the time. A C-statistic of 0.50 indicates the predictor domain is no more predictive than random chance. See S3 Appendix for all models used to identify statistically significant correlates and for the variables included in each model but omitted from this figure because they were not statistically significant.

The statistically significant correlates of adherence within each domain are presented in Figure 2 (see S3 Appendix for detailed regression models). There are a number of correlates that move in expected directions. For example, perceived risk of death and the perception of masks as beneficial to health are positively associated with adherence level. Trust in national and left-leaning media and rates of county-level unemployment are both positively associated

with adherence, while affiliation with the Republican party, in-person work, and rates of county-level support for President Trump in the 2020 election are negatively associated with adherence.

There are a few significant correlates that move in unexpected directions. For example, there is a significant negative association between perceived risk of COVID infection and adherence and a significant positive association between inability to afford a mask and adherence. These associations may be due to demographic or other confounders not included in the health beliefs domain. We account for confounders in our meta-regression analysis.

Meta-regression results can be found in Figure 3, which plots the percentage of each domain's predictive value that is retained after controlling for all other domains. On the x-axis, each domain is plotted in order of its C-statistic to denote the domain's predictive value prior to controls. See S3 Appendix for odds ratios and standard errors from the regression model.

Fig 3: Predictive Value Retained in Meta-Regression Model, by Predictor Domain and C-Statistic

Note: This figure reports the percentage of each domain's predictive value (measured as the predicted probability of high adherence associated with the modification of that domain) that is retained after controlling for all other domains. For example, health beliefs retain 94% of their predictive value after adjusting for other domains, which means that 6% of the predictive value of health beliefs is explained away by other domains. On the x-axis, each domain is plotted in order of its C-statistic. C-statistics represent the domain's original predictive performance prior to controls. A C-statistic of 0.50 is the same as random chance.
*These predictors have the same C-statistic as political affiliation (0.64).

Results show that health beliefs retain 94% ($p<0.001$) of their predictive value, which means only 6% is explained away by other domains. This percentage-retained is much higher than the percentage retained by other individual and contextual domains. After health beliefs,

interpersonal context retains the highest percentage of its predictive value, at 87% (p<0.001), although its predictive performance was quite poor, as indicated by its C-statistic of 0.59. Community context, work context, and socio-demographic characteristics retain 52% (p<0.001), 34% (p<0.001), and 23% (p<0.001) of their predictive value after adjustment, respectively. Most notably, political affiliation retains 6% (p=0.166) of its predictive value and informational trust retains 0% (p=0.992), meaning essentially all of the predictive value of political affiliation and informational trust is explained by their relationship to other domains, mainly health beliefs.

The C-statistic for the entire meta-model (0.82) is not much higher than the C-statistic generated by health beliefs alone (0.80). This suggests individual and contextual domains contribute very little to the prediction of adherence above and beyond their relationship to health beliefs.

Discussion

Two key findings emerge from this analysis of protective behavior in private social settings. First, patterns of adherence fall into three categories marked by high, moderate, or low levels of social-distancing and masking behavior, with under half (44%) of respondents exhibiting consistently high levels of adherence. Second, in support of prior research on the HBM,[24] health beliefs are the single strongest predictor of adherence to protective social behavior. Alone, they predict the largest share of observed adherence and they have the strongest direct, or unmediated, relationship to adherence.

Compared to health beliefs, all other contextual and individual-level predictors have either poor predictive power or poor primarily indirect associations. Political affiliation and

informational trust, for example, are moderately predictive of behavioral adherence but their relationship to behavior is entirely explained by their relationship to other predictor domains, mainly health beliefs. In line with prior research,[25,26] these results suggest political affiliation and informational trust *do* influence protective behavior insofar as they influence beliefs.

A few qualifications to this analysis should be acknowledged. First, our results describe protective behavior in *private interpersonal settings*. We focus on these settings because they have been hotspots for the community spread of COVID-19[27,28] but the private nature of these settings may explain why the effect of community context in our model is primarily mediated by individual-level factors. In public settings, we might expect a different pattern than this to unfold, where communities directly influence behavior without necessarily influencing beliefs.

Second, this analysis includes a limited set of measures of organizational and interpersonal context. While we were able to account for the size of respondents' social networks in this study, we could not account for network composition, although we expect this to be correlated with community and socio-demographic characteristics. Furthermore, while we were able to account for work conditions, we could not account for other organizational contexts in which respondents are embedded. Third, our results reflect average patterns and predictors of adherence to protective behavior during the COVID-19 pandemic, which may or may not mirror patterns and predictors at a single point in time or in other public health emergencies.

Conclusion

The results of this study have implications both for public health strategies to mitigate the spread of COVID-19 and for the general challenge of disease mitigation in public health emergencies. Results show clearly that health beliefs drive adherence to protective measures in private social settings; other individual-level and contextual factors carry only modest predictive value or their relationship to behavior is largely mediated by their relationship to health beliefs. It is important not to conclude from this, however, that indirectly predictive factors, like informational trust or community context, are unimportant. To the contrary, our results suggest these factors *do* matter, primarily *because* of their relationship to health beliefs. This means local communities and trusted messengers may have minimal impact on private protective behavior if their messages are misaligned with, or fail to change, people's beliefs. Put simply, a successful mitigation strategy for private social settings depends up on the ability of trusted sources, messengers, policymakers, and communities to influence health beliefs.

Acknowledgements

We thank Rashmi Shetgiri, MD, MSHS, MSCS, and Paul Simon, MD, MPH, of the Los Angeles County Department of Public Health as well as Jeanne R. Delgado, MD, MPH, of the National Clinician Scholars Program, Division of General Medicine & Health Services Research, at the University of California, Los Angeles, for their input on study design and interpretation of results.

Data collection for this study was supported by the University of Southern California, the Bill & Melinda Gates Foundation, and federal funds from the National Institute on Aging (grant 5U01AG054580-03) and the National Science Foundation (grant 2028683). The sponsors had no role in study design; collection, analysis, and interpretation of data; writing the report; or the decision to submit the report for publication

References

1. Crawford FW, Jones SA, Cartter M, et al. Impact of close interpersonal contact on COVID-19 incidence: Evidence from 1 year of mobile device data. *Science Advances*. 8(1):eabi5499. doi:10.1126/sciadv.abi5499

2. Darling J, Thomas K, Kapteyn A, Theys N, Cassil A. *Most U.S. Adults Wear Masks - Inconsistently*. USC Dornsife Center for Economic and Social Research; 2021. file:///Users/kylathom/Downloads/CESR_Policy_Brief_Mask_Wearing_January_21_2021%20(1).pdf

3. Rosenstock IM. The Health Belief Model and Preventive Health Behavior. *Health Education Monographs*. 1974;2(4):354-386. doi:10.1177/109019817400200405

4. Rosenstock IM. Why People Use Health Services. *Milbank Q*. 1966;44(3):94-124. doi:10.1111/j.1468-0009.2005.00425.x

5. Bish A, Michie S. Demographic and attitudinal determinants of protective behaviours during a pandemic: A review. *British Journal of Health Psychology*. 2010;15(4):797-824. doi:10.1348/135910710X485826

6. Jørgensen F, Bor A, Petersen MB. Compliance without fear: Individual-level protective behaviour during the first wave of the COVID-19 pandemic. *British Journal of Health Psychology*. 2021;26(2):679-696. doi:10.1111/bjhp.12519

7. Schoeni RF, Wiemers EE, Seltzer JA, Langa KM. Association Between Risk Factors for Complications From COVID-19, Perceived Chances of Infection and Complications, and Protective Behavior in the US. *JAMA Network Open*. 2021;4(3):e213984. doi:10.1001/jamanetworkopen.2021.3984

8. Carpenter CJ. A Meta-Analysis of the Effectiveness of Health Belief Model Variables in Predicting Behavior. *Health Communication*. 2010;25(8):661-669. doi:10.1080/10410236.2010.521906

9. Janz NK, Becker MH. The Health Belief Model: A Decade Later. *Health Education Quarterly*. 1984;11(1):1-47. doi:10.1177/109019818401100101

10. Wong LP, Alias H, Wong PF, Lee HY, AbuBakar S. The use of the health belief model to assess predictors of intent to receive the COVID-19 vaccine and willingness to pay. *Human Vaccines & Immunotherapeutics*. 2020;16(9):2204-2214. doi:10.1080/21645515.2020.1790279

11. Geana MV, Rabb N, Sloman S. Walking the party line: The growing role of political ideology in shaping health behavior in the United States. *SSM - Population Health*. 2021;16:100950. doi:10.1016/j.ssmph.2021.100950

12. Szilagyi PG, Thomas K, Shah MD, et al. Likelihood of COVID-19 vaccination by subgroups across the US: post-election trends and disparities. *Human Vaccines & Immunotherapeutics*. 2021;17(10):3262-3267. doi:10.1080/21645515.2021.1929695

13. Wynen J, Op de Beeck S, Verhoest K, et al. Taking a COVID-19 Vaccine or Not? Do Trust in Government and Trust in Experts Help us to Understand Vaccination Intention? *Administration & Society*. Published online February 14, 2022:00953997211073459. doi:10.1177/00953997211073459

14. Szilagyi PG, Thomas K, Shah MD, et al. The role of trust in the likelihood of receiving a COVID-19 vaccine: Results from a national survey. *Preventive Medicine*. 2021;153:106727. doi:10.1016/j.ypmed.2021.106727

15. Dohle S, Wingen T, Schreiber M. Acceptance and Adoption of Protective Measures During the COVID-19 Pandemic: The Role of Trust in Politics and Trust in Science. *Social Psychological Bulletin*. 2020;15(4):1-23. doi:10.32872/spb.4315

16. Sheeran P. Intention—Behavior Relations: A Conceptual and Empirical Review. *European Review of Social Psychology*. 2002;12(1):1-36. doi:10.1080/14792772143000003

17. Armitage CJ, Conner M. Social cognition models and health behaviour: A structured review. *Psychology & Health*. 2000;15(2):173-189. doi:10.1080/08870440008400299

18. Stokols D. Establishing and maintaining healthy environments: Toward a social ecology of health promotion. *American Psychology*. 1992;47(1):6-22. doi:10.1037/0003-066X.47.1.6

19. Stokols D. Translating Social Ecological Theory into Guidelines for Community Health Promotion. *Am J Health Promot*. 1996;10(4):282-298. doi:10.4278/0890-1171-10.4.282

20. Golden SD, Earp JAL. Social Ecological Approaches to Individuals and Their Contexts: Twenty Years of Health Education & Behavior Health Promotion Interventions. *Health Educ Behav*. 2012;39(3):364-372. doi:10.1177/1090198111418634

21. Kleitman S, Fullerton DJ, Zhang LM, et al. To comply or not comply? A latent profile analysis of behaviours and attitudes during the COVID-19 pandemic. *PLOS ONE*. 2021;16(7):e0255268. doi:10.1371/journal.pone.0255268

22. Kapteyn A, Angrisani M, Bennett D, et al. Tracking the Effect of the COVID-19 Pandemic on the Lives of American Households. *Survey Research Methods*. 2020;14(2):179-186. doi:10.18148/srm/2020.v14i2.7737

23. Sternthal MJ, Slopen N, Williams DR. RACIAL DISPARITIES IN HEALTH. *Du Bois Rev*. 2011;8(1):95-113. doi:10.1017/S1742058X11000087

24. Tong KK, Chen JH, Yu EW yat, Wu AMS. Adherence to COVID-19 Precautionary Measures: Applying the Health Belief Model and Generalised Social Beliefs to a Probability Community

Sample. *Applied Psychology: Health and Well-Being*. 2020;12(4):1205-1223. doi:10.1111/aphw.12230

25. Ghio D, Lawes-Wickwar S, Tang MY, et al. What influences people's responses to public health messages for managing risks and preventing infectious diseases? A rapid systematic review of the evidence and recommendations. *BMJ Open*. 2021;11(11):e048750. doi:10.1136/bmjopen-2021-048750

26. Favero N, Jilke S, Wolfson JA, Xu C, Young MM. Messenger effects in COVID-19 communication: Does the level of government matter? *Health Policy OPEN*. 2021;2:100027. doi:10.1016/j.hpopen.2020.100027

27. Colorado 11_1_20 - DocumentCloud. Accessed June 15, 2022. https://www.documentcloud.org/documents/7298365-Colorado-11-1-20.html

28. At dinner parties and game nights, casual American life is fueling the coronavirus surge as daily cases exceed 150,000. *Washington Post*. https://www.washingtonpost.com/health/2020/11/12/covid-social-gatherings/. Accessed June 15, 2022.

S1 Appendix

S2 Table

S3 Appendix

 Click here to access/download;Figure;Fig1.pdf

Fig 1: Modified Conceptual Framework and Measures, Based on Social-Ecological and Social-Cognitive Models

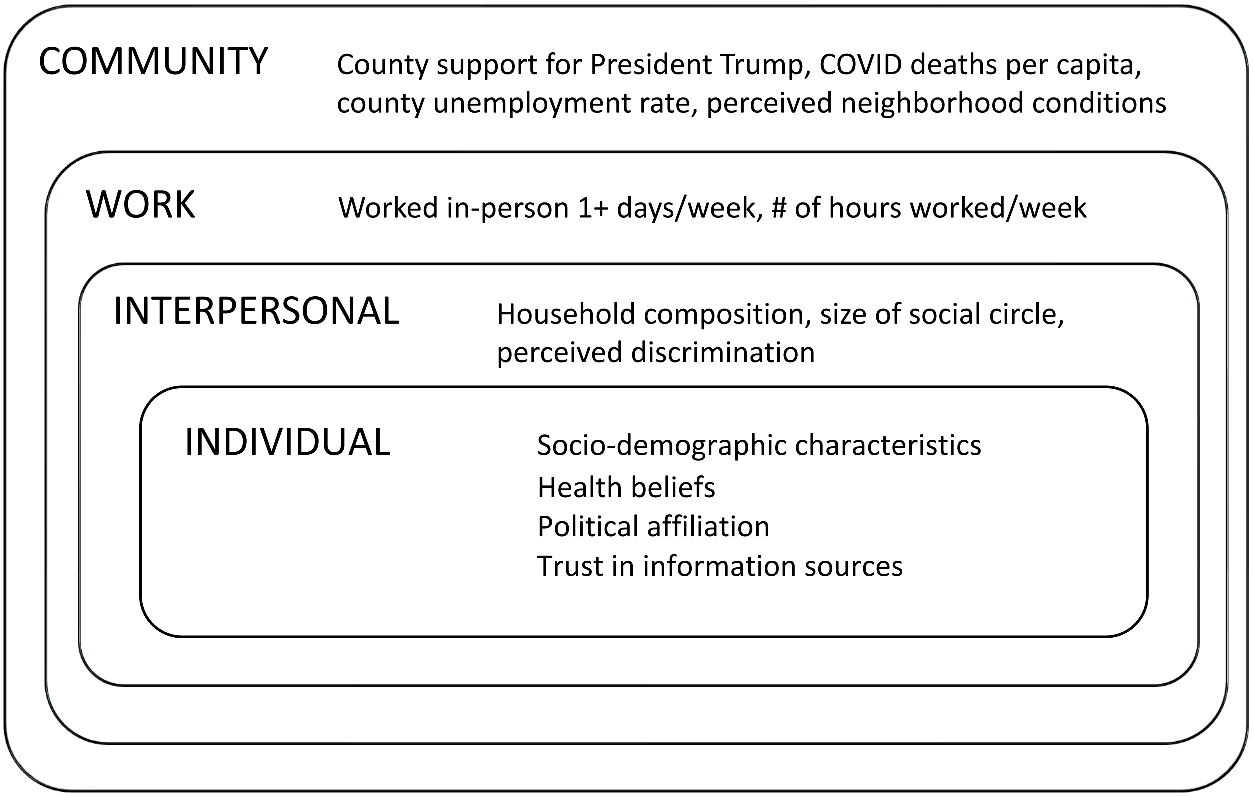

Fig 2 Click here to access/download;Figure;Fig 2.pdf

Fig 2: Predictors of Adherence to Protective Social Behavior

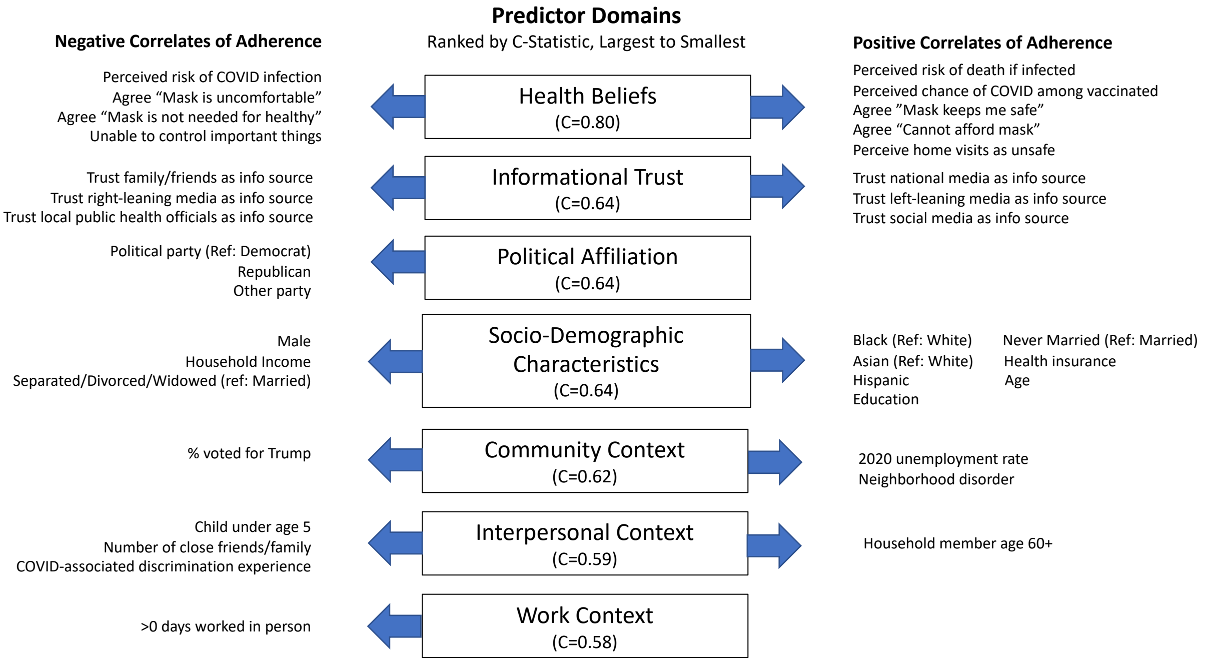

Fig 3: Predictive Value Retained in Meta-Regression Model, by Predictor Domain and C-Statistic

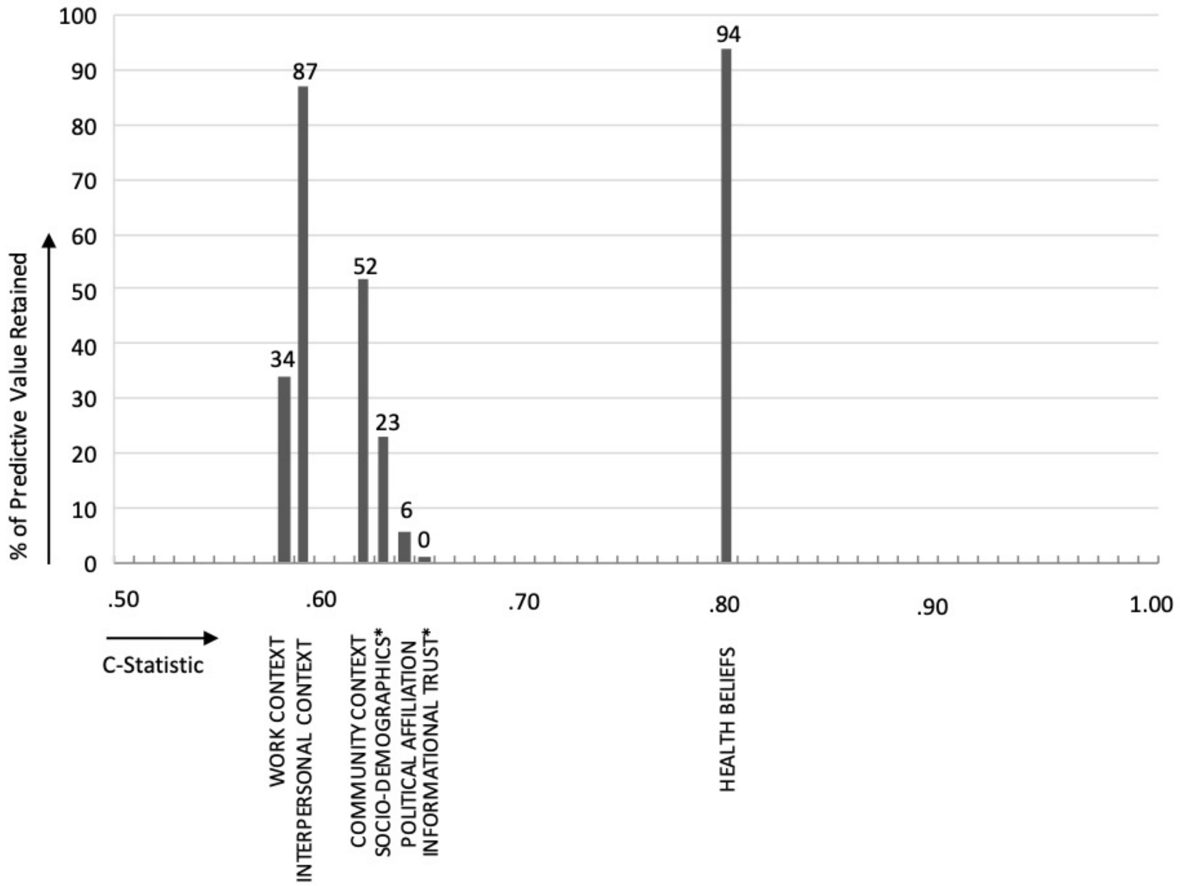

S1 Appendix

Click here to access/download
Supporting Information
S1 Appendix.docx

S2 Table

Click here to access/download
Supporting Information
S2 Table.docx

Click here to access/download
Supporting Information
S3 Appendix.docx
